# Biological aging of two innate behaviors of *Drosophila melanogaster*: Escape climbing versus courtship learning and memory

**DOI:** 10.1371/journal.pone.0293252

**Published:** 2024-04-09

**Authors:** Jessica Thiem, Maria Viskadourou, Alexandros Gaitanidis, Dimitrios J. Stravopodis, Roland Strauß, Carsten Duch, Christos Consoulas

**Affiliations:** 1 Institute of Developmental Biology and Neurobiology, Johannes Gutenberg University Mainz, Mainz, Rhineland-Palatinate, Germany; 2 Laboratory of Experimental Physiology, Medical School, National and Kapodistrian University of Athens (NKUA), Athens, Greece; 3 Section of Cell Biology and Biophysics, Department of Biology, School of Science, National and Kapodistrian University of Athens (NKUA), Athens, Greece; Biocenter, Universität Würzburg, GERMANY

## Abstract

Motor and cognitive aging can severely affect life quality of elderly people and burden health care systems. In search for diagnostic behavioral biomarkers, it has been suggested that walking speed can predict forms of cognitive decline, but in humans, it remains challenging to separate the effects of biological aging and lifestyle. We examined a possible association of motor and cognitive decline in *Drosophila*, a genetic model organism of healthy aging. Long term courtship memory is present in young male flies but absent already during mid life (4–8 weeks). By contrast, courtship learning index and short term memory (STM) are surprisingly robust and remain stable through mid (4–8 weeks) and healthy late life (>8 weeks), until courtship performance collapses suddenly at ~4.5 days prior to death. By contrast, climbing speed declines gradually during late life (>8 weeks). The collapse of courtship performance and short term memory close to the end of life occur later and progress with a different time course than the gradual late life decline in climbing speed. Thus, during healthy aging in male *Drosophila*, climbing and courtship motor behaviors decline differentially. Moreover, cognitive and motor performances decline at different time courses. Differential behavioral decline during aging may indicate different underlying causes, or alternatively, a common cause but different thresholds for defects in different behaviors.

## Introduction

In the face of increasing life expectancy in modern societies, aging-related decline in cognitive and motor performance becomes an increasing burden for health and social care systems [1, 2]. A fundamental prerequisite toward healthy aging is a better mechanistic understanding how the process of aging manifests in malfunction of the nervous system, but aging is a multi-causal, highly variable and individual process. In all animals, from worms [3] to flies [4] and humans [5, 6], some individuals reach high ages without major impairments and are commonly referred to as wellderlies, whereas others suffer from prolonged periods of cognitive and/or motor decline. On the molecular level the nine main causes of aging are genomic instability, telomere attrition, epigenetic alterations, loss of proteostasis, deregulated nutrient sensing, mitochondrial dysfunction, cellular senescence, stem cell exhaustion, and altered intercellular communication [7]. However, the resulting impairments on the nervous system and behavioral levels are diverse, and it is often difficult to separate the functional consequences of biological aging, diseases, and life-style. Some authors have proposed that one common cause underlies diverse aging phenotypes in the human nervous system, and this has been referred to as a unifying cause of aging [8]. In support of this, positive correlations between specific aspects of motor function and cognitive decline have been provided [9, 10] during human aging. In particular, in older adults, walking speed decrease is a good predictor of cognitive decline, but the reverse does not seem to be the case [11, 12]. On the other hand, ample evidence across species shows that different types of neurons and neural circuits [13, 14], as well as different brain parts are differentially vulnerable to age-related and neurodegenerative decline [15]. Within neurons, the synaptic compartment seems to be the most vulnerable one [15]. Consequently, different cellular and circuit malfunctions have been attributed to underlie different age-related behavioral impairments. For example, in non-primate monkeys working memory decline has been related to high vulnerability of thin spines in prefrontal cortex [16], whereas loss of navigational ability has been attributed to reduced plasticity in place and grid cell circuitry [17]. By contrast, the cellular causes for aging-related reduced locomotion speed that is apparent across species [9, 18–20] remain largely unclear. Is there a unifying cause for multiple age-related impairments, and can some predict the occurrence of others? To start addressing this question, longitudinal studies of multiple aging-related nervous system malfunctions are needed [9].

As entry point toward addressing this question in the invertebrate genetic model system, *Drosophila melanogaster*, we investigate age-related decline of two well-studied innate behaviors in parallel, namely frustration learning during courtship [21] and escape climbing behavior [22]. *Drosophila* has the advantage of a relatively short life-span of 60 to 90 days under laboratory conditions [4]. First, this allows longitudinal studies within a few months. Second, neurodegenerative diseases are modeled in *Drosophila* upon introduction of human disease factors, but do not natively exist, so that longitudinal assessment can be conducted with healthy flies. This circumvents the problem that age-related changes in cognitive and physical functioning often result from interactions between the normal aging process and diseases with unknown onset, which makes their effects often indistinguishable [23]. In addition, the genetic power of the *Drosophila* model system has advantaged to address the molecular mechanisms of aging-related cognitive and motor decline, and the relative simplicity of the nervous systems ~100.000 neurons) may help identifying common or diverse cellular causes for cognitive and motor decline.

We have previously described the patterns of normal *Drosophila* motor aging and found that impairment onset, duration, and severity are highly variable and unpredictable [4], just as is the case in mammals. Moreover, flies can remain healthy until a few hours prior to death (wellderlies), or suffer from multiple days of combinations of different motor impairments (illderlies), again phenotyically similar to what has been reported for mammals [24, 25], including humans [26]. On the other hand, many studies have used *Drosophila* to investigate memory decline in old flies [27–31]. However, knowledge on the relationship between the time course of motor and cognitive decline is sparse. We unravel the patterns of age-related decline in courtship learning and memory in parallel with that of locomotion decline to test whether one can predict the other, whether both occur in synchrony, or whether both manifest independently from each other.

## Methods

### Animals, rearing conditions

Oregon-R (strain: # 5, Bloomington fly stock center) wild type flies were reared at 25°C and 70% relative humidity under a 12-h/12-h Light/Dark cycle. (rearing conditions:24±1 C°, 70% humidity; conditions during experimental trials:21±2 C° 60% humidity). Diet was based on corn flour-yeast agar medium containing 0.75% (w/v) agar, 4.5% (w/v) dry yeast, 3.5% (w/v) corn meal, 5.5% (w/v) Sucrose, 0.4% (v/v) Propionic acid, 2.5% (v/v) nipagen diluted in 10% absolute ethanol.

### Physical functioning and reactivity assay applied daily from 40 day of age until death

The Zeitgeber was a 12h light/dark cycle with light-on at 8am and light-off at 8pm with testing once/day at 10am (light) in all males from the age of 60 days until death. Escape performance was tested in individual flies in their food vials by gently banging the vial on the counter. We applied a personalized assay described in [4]. In brief in this assay a fly is aroused by gentle but abrupt tapings of the vial, that releases walking or/and climbing behaviors and thus allowing to assess the physical status and diagnose walking impairments. The resulting physiological status criteria are described in Figs 3 and in 4.

### Startle escape assay

Oregon-R males were singly isolated in falcon vials and left undisturbed for 30 minutes, enough time for acclimation in the new environment. Then each fly received three light stimuli (displacement of the fly at the bottom of the vial by tapping or flipping the vial) followed by six moderate stimuli (1 s vortexing) in two rounds. Finally, each fly received a combination of ~20 mixed stimuli (flipping, banking and vortexing of the vial) in a fast pace. This fast delivery of different stimuli caused, at least one fierce escape response during which the fly performs close to its ability. We measured the time-to-climb a 6 cm vertical distance on the wall of the vial within 10 seconds. Failure was considered the unsuccessful effort of completing the task within 10 s. The startle assay was used to test the fitness of the flies in our longitudinal study. Both courtship motor performance as well as courtship learning require the ability to initiate and maintain a motor behavior. Successful performance in the startle assay means that the fly has initiated escape climbing upon the startle stimulus and maintain escape climbing at least until it has reached a height of 6 cm at the wall of the vial. Only flies that could complete the task were admitted to longitudinal study. We did not further distinguish between escape climbing initiation and maintenance.

### Courtship and courtship conditioning assay

Courtship assays were performed in mating chambers (10 mm diameter, 5 mm depth) and recorded with digital camcorders (Sony HDR-XR260). Flies were collected on the day of eclosion and kept in a 25°C incubator with a 12/12 hour L/D cycle. Naive males were kept in individual test tubes and target females and males for mating were store in small groups. The total amount of time a male was engaged in courtship activity with an unanesthetized target female (trainer or tester) during a test period of 10 min or until successful copulation occurred was scored. Overall, we followed the experimental procedures described in [32]. For courtship condition assays, a single test male was placed in the chamber with a mated female (trainer) for one hour. The first and last 10 minutes were recorded and analyzed. After a 5 min isolation period, the trained male faced a tester (unanesthetized virgin female) and the 10 first minutes were recorded. A sham-trained male was kept alone in the courtship chamber for one hour and paired with a tester female for 10 min. Thus, for each 10-min recording, we calculated the courtship index (CI), CI initial, CI final, CI test, and CI sham. For long-term memory we followed the courtship conditioning assay protocol as described in [33]. The learning index is the ratio of the courtship level in the final 10 min of the training (CI final) to that of the initial 10 min (CI initial). The memory index is the ratio CI test/the mean of CI sham. A memory index close to 1 indicates that there is no memory because the courtship level of the trained males is similar to that of the sham-trained males.

### Statistical analysis

All measurements were tested for Gaussian distribution by D’Agostino & Pearson omnibus normality test. When the data was following Gaussian distribution, the unpaired t-test was used. For comparison of two samples with data not following Gaussian distribution the two-tailed Mann—Whitney-U tests (95% confidence level, statistical significance P<0.05) was performed. For comparison of more than two groups, Kruskal—Wallis ANOVA and Dunn’s post-hoc test for multiple comparisons (statistical significance P<0.05) was used. The measurements were presented as means with SEM in scatter plots with bars. Outliers were not shown. Significant differences were accepted at p< 0.05. All statistical analyses in this study were performed using GraphPad Prism version 6.00 for Windows, (GraphPad Software, La Jolla, CA, USA).

The data measurements for all figures are provided at https://doi.org/10.5281/zenodo.10629747

## Results

We used two well studied innate behaviors (escape climbing and courtship) to uncover the age-dependent changes in physical and cognitive functioning of male flies during mid life (4–8 weeks) and late life (>10 weeks). First, we examined the physical status of mid life and late life males in the escape climbing assay. Flies housed in cylindrical vials can be startled by mechanical stimuli, such as banging of the vial onto the top of the counter. In response to this disturbance the animal immediately escapes by climbing up the wall of the vial, obeying its innate negative geotaxis tendency. Less common is escape by jumping, or flight. Previous studies have demonstrated that climbing performance gradually decays with age [22], and that climbing ability is a good proxy for physiological age, whereas climbing impairment is a predictor of death [4].

**Fig 1:**
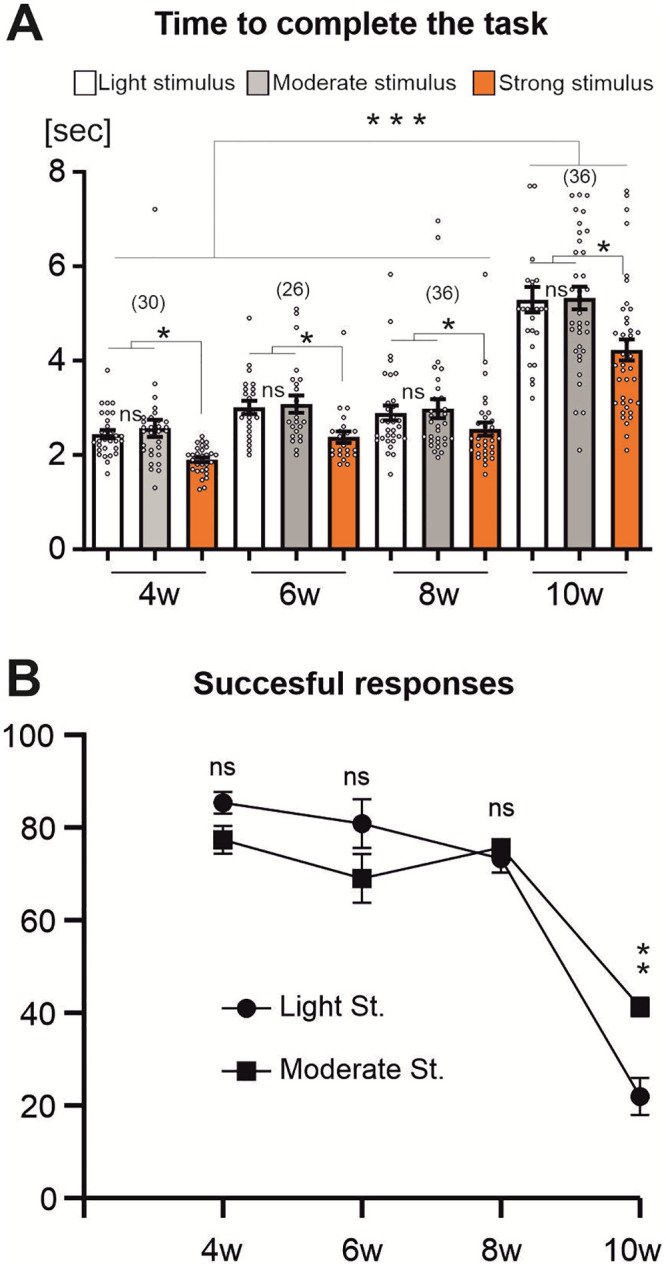
Climbing performance remains stable in mid life but decreases during late life. **(A)** Male flies of different ages were individually tested for their performance in climbing a 6 cm vertical distance after having been startled with different stimulation regimes. The strongest stimuli caused the fastest responses at all ages. **(B)** Successful responses (completion of the 6 cm climbing task within 10 s) remain unaltered between 4 and 8 weeks of age (mid life) but decline after the 8^th^ week (late life). (One-Way ANOVA, Kruskal-Wallis multiple comparison test, * p<0.05, ** p<0.01, *** p<0.001).

In preliminary studies we noticed that escape climbing is scalable with the arousal level of the individual and the intensity of stimulation. Arousal level was controlled for as good as possible by treating all individuals identically for 30 minutes before testing (see methods). To account for stimulus intensity, we used three different stimulation regimes (see methods) for measuring escape performance (time to complete climbing a 6 cm vertical distance) in individual male flies of four different age groups (4, 6, 8, 10 weeks). We found that the strongest stimulation induced the fastest climbing responses across all ages tested (Fig 1A). Independently of the type and intensity of the stimulation regime, climbing performance remains constant during mid life (4–8 weeks of age), but decreases significantly between the 8^th^ and 10^th^ week (Fig 1A). Similarly, the percentage of successful responses (completion of the task within 10 s) drops from ~80% in 8 weeks old flies to 20%–40% (depending on stimulus strength) in 10 weeks old flies (Fig 1B). Thus, at a critical time after the eighth week of age, males become slower and fail more often to complete the task, but these flies are still capable of performing escape climbing.

**Fig 2:**
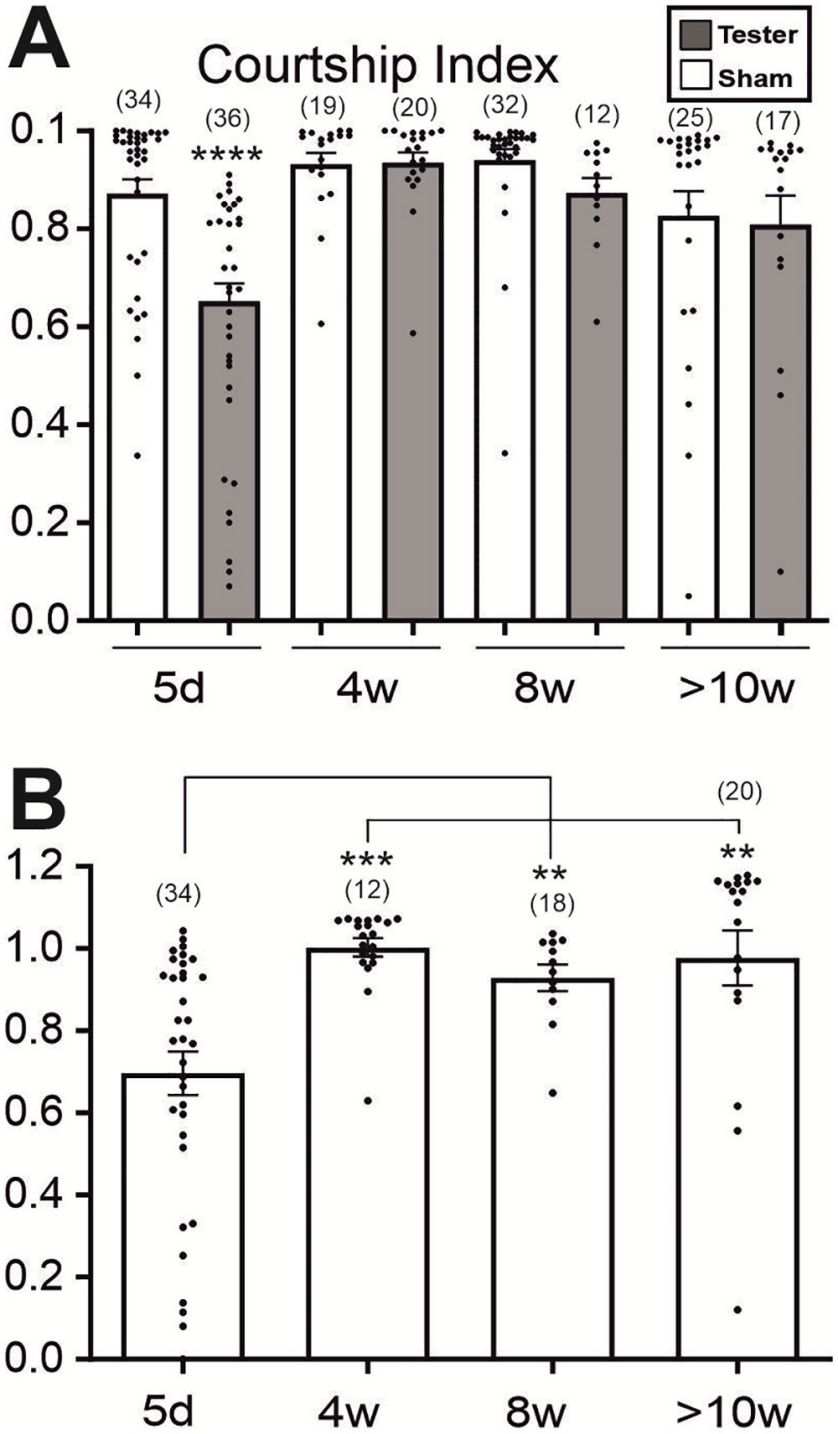
Courtship LTM is absent during mid and late life. **(A)** When presented with a virgin female, courtship indices of the Tester (males presented with a mated female on the previous day) and Sham males (naïve males) differ in young flies (5d), thus indicating courtship LTM. However, Tester and Sham courtship indices are not different during mid and late life. Courtship index was estimated as the percentage of male courtship within a 10 minutes time window. Tested males first courted towards a mated female for 8 hours, were then isolated for 24 hours, and finally courted against a virgin tester female. **(B)** LTM (24 hours) courtship memory index in mid and late life. Note that the LTM index approaches 1 at 4 weeks and all older ages tested, indicating absence of LTM from 4 weeks on. (T-test for A, One-Way Anova, Kruskal-Wallis multiple comparison test for B. ** p<0.01, *** p<0.001, **** p<0.0001).

Since we aim to unravel the patterns of age-dependent escape climbing decay and relate this to courtship learning and memory tasks, longitudinal testing of individual flies is necessary. Flies develop individual locomotor and behavioral disabilities prior to their natural death independently of the age they die [4]. This pre-death morbidity period can vary from few hours to several days, has an unpredictable onset, and requires daily testing to be traced. Moreover, for measuring courtship learning and memory indexes daily, it is imperative to first investigate to what extent 24 hours long term memory (LTM) traces from the previous day affect the outcome of the next day’s test. We therefore estimated the 24 hours courtship LTM in males of 4, 8, and 10 weeks of age (Fig 2). All males were fit individuals capable of performing escape climbing. Courtship index was similarly high in both, the tested males (confronted with a virgin female, but subjected to rejection by a mated female on the previous day) and the sham males (not previously frustrated, Fig 2A), and thus the long-term memory (LTM) index approaches 1 at all ages of 4 weeks and older (Fig 2B). This shows that no courtship memory is produced by this conditioning with a previously mated female target at any of the mid to advanced ages tested. However, courtship LTM is present in young flies (Fig 2B), as previously shown by others [34]. Ensuring that males do not remember their frustration with the mated female next day, the precondition for daily testing of courtship conditioning and escape climbing in longitudinal studies is fulfilled.

**Fig 3:**
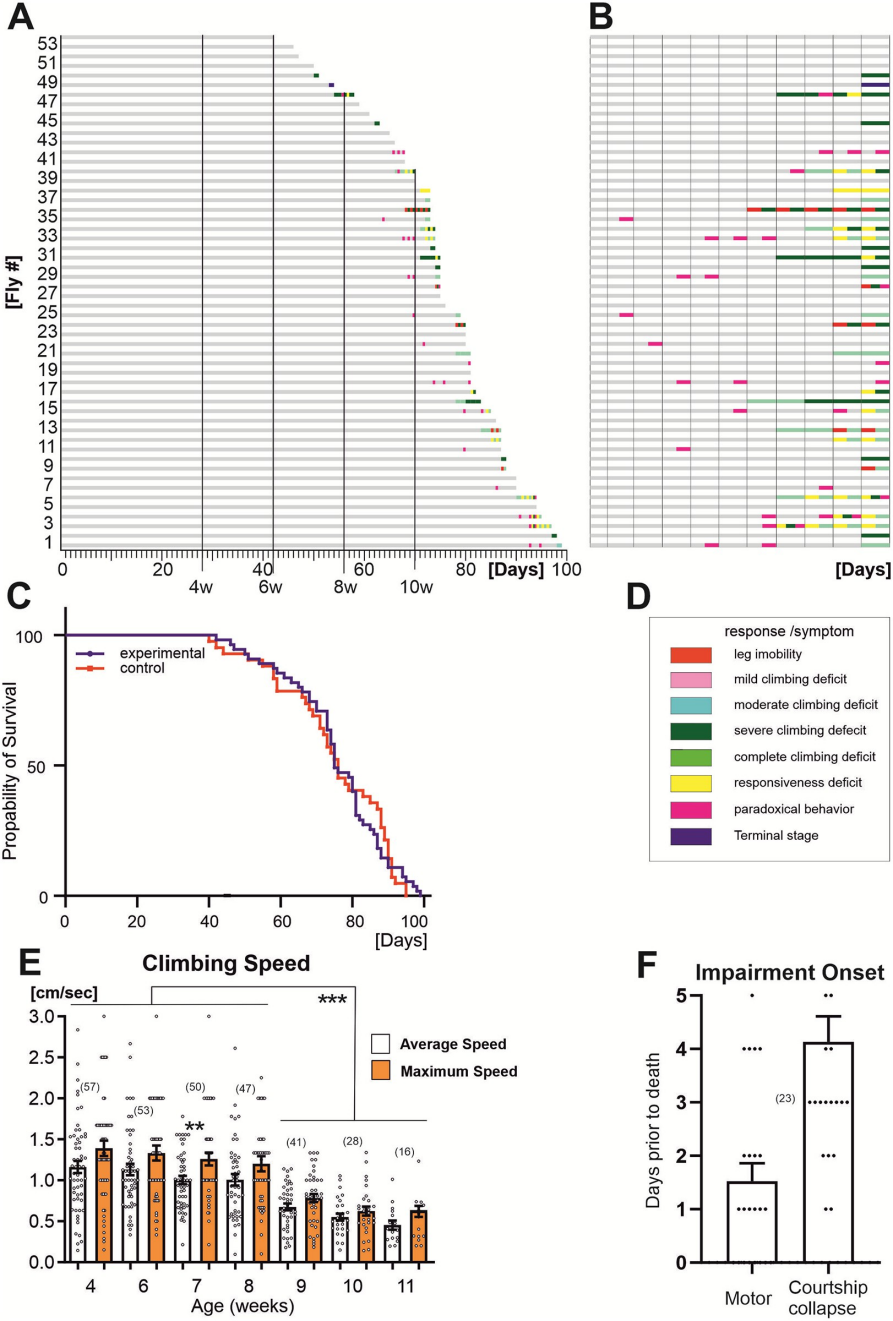
Late life pathophysiology of locomotor behavior. **(A)** Life history chart for 53 male Oregon-R flies tested daily and individually in the startle assay from the age of 60 days until death. Gray bars indicate health-span and colored bars disabilities of different categories as defined in ([4]; see also D). 39.6% (21 out of 53) of the flies show no sign of impairment until the last day of life. All flies, with or without disabilities during the last day of life, undergo a dramatic functional collapse (terminal stage) characterized by immobility, unresponsiveness and finally death within few hours [4]. Since terminal stage lasts only few hours and is followed by death it was not detected in the majority of flies by testing once a day. **(B)** Time enlargement of the last 10 days for all flies. Note that days are separated by vertical lines and combinations of colored bars show co-morbidities. **(C)** Survival curves for the tested population in **A** and non-tested control flies are not significantly different [Log-rank (Mantel-Cox) test; X^2^ = 0.001431, p = 0.9698]. (**D**) Color-code for behavioral impairments as defined in [4]. (**E**) Maximum climbing speed drops significantly between the 8^th^ and 9^th^ week. (**F**) Severe reduction (>90%) in male courtship occurs on average 4 days prior to death, 2.5 days earlier than the onset of motor impairments. (One-Way ANOVA, Kruskal-Wallis multiple comparison test for E; T-test for F. ** p<0.01, *** p<0.001).

We next performed a longitudinal study with 53 males to study motor and cognitive performance, measured in climbing and courtship conditioning assays (Fig 3). All animals for which daily fitness testing detected pre-death morbidity, such as an inability to climb, were not tested on subsequent days. Therefore, all animals were tested until they reached the pre-death impairment period (Fig 3A and 3B). Twenty-five of these flies reached ages >10 weeks without impairment and were tested daily until pre-death morbidity. In the same experimental run, another population of single males (N = 42) was not tested but scored for survival (control). Experimental testing did not reduce lifespan (Fig 3C). An event history chart summarizes health-span (Fig 3A, gray bars), impairment-span (Fig 3A and 3B, colored bars), and lifespan for all 53 flies (Fig 3A). The different physiological status conditions depicted by the colored bars (Fig 3A and 3B) have previously been described in detail [4]. Briefly, normal escape responses (gray bars) include climbing to the top of the vial, or jumping, or flying. Leg joint immobility is a joint defect where the leg remains permanently extended or retracted. Flies that exhibit mild climbing defect are usually slow, often with a leg defect, but can climb to the top. Moderate climbing defects characterize flies that can climb but no higher than 1/3 of the vial’s height. Severe and complete climbing defects describe flies that can barely climb before falling off the wall or cannot climb at all, respectively. Paradoxical behavior is a transient state during which the fly performs unexpected responses to the stimulation (e.g. walking in circles, aggressive behavior, coma-like periods followed by full recovery, etc). The responsiveness deficit relates to a weak reaction to stimulation and an almost absent startle response. Terminal stage is the last short phase of life during which flies become supine and immobile, exhibit leg tremor, rhythmical abdominal bending, and proboscis extension/retraction movements. Startle response impairments (color code for impairment type, Fig 3D) were detected in 60.4% of all individuals on average 1.5 days prior to death (Fig 3F). The remaining 39.6% were devoid of pathological phenotypes (Fig 3A) and responded normally to the stimulation by climbing, jumping, or flying, until 1 day prior to death (Fig 3A and 3B). As previously shown [4], even these “wellderly” flies eventually develop impairments for some hours prior to death. We measured the climbing speed of all fit males up to 11 weeks of age (Fig 3E). Maximum and average climbing speed are lower in mid aged flies than reported for young flies [19], remain unaltered throughout mid life (4–8 weeks), but decline significantly between the 8th and 9th week (Fig 3E). This pattern of decay confirmed our finding from the cross-sectional study (Fig 1).

**Fig 4:**
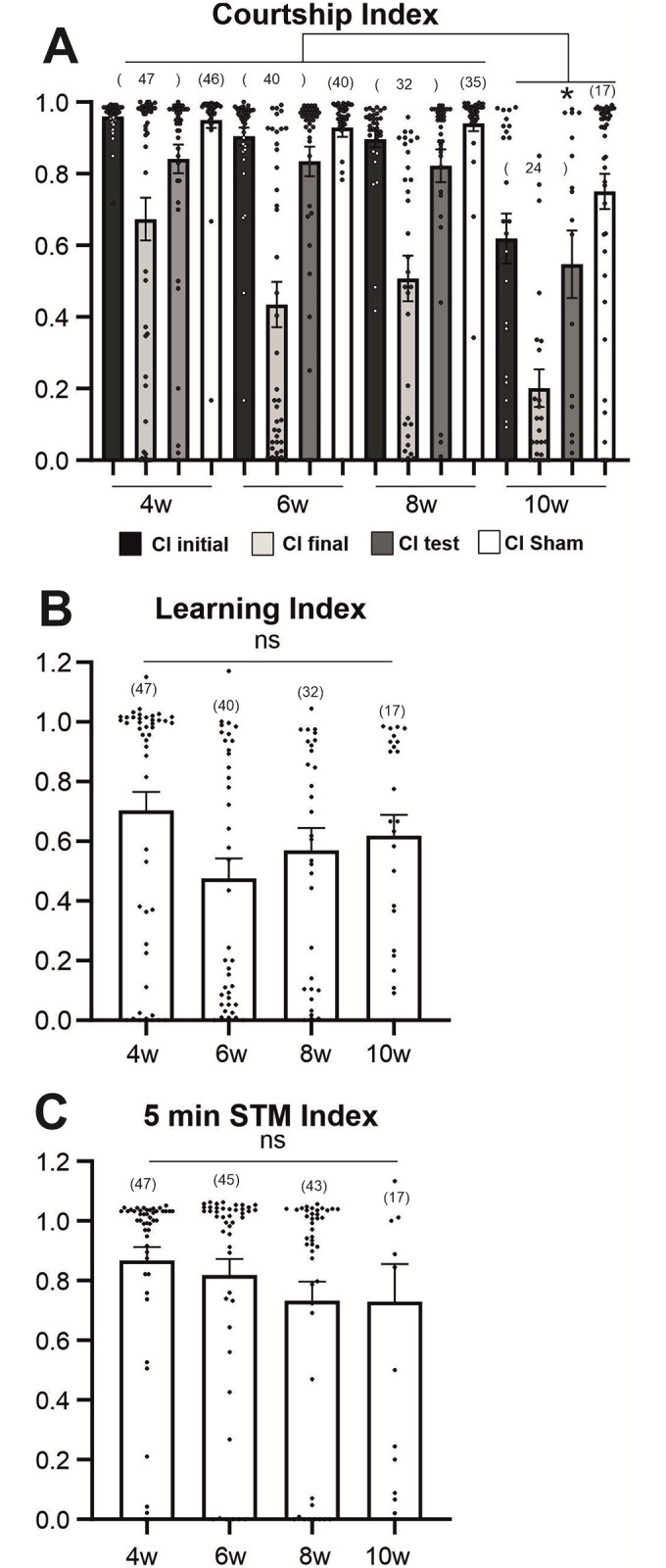
Male courtship index decays in old flies, but courtship learning and (STM) remain stable. (**A**). Courtship Index is estimated as the percentage of male courtship within a 10 min time window. Courtship conditioning assays were performed with the males of different ages (4, 6, 8, and 10 weeks of age). First the trained male courts against a mated female trainer for one hour [recorded:10 first min with trainer (CI initial), 10 last mins with trainer (CI final)]. Next, after 5 min isolation the trained male is presented to a young virgin female tester [recorded:10 first mins with the virgin tester (CI test)]. As a control, age-matched, non-trained males (Sham) are presented with a virgin female. (**B**) The learning index is the ratio of the courtship indices during the final 10 min of the training (CI final) and that of the initial 10 min (CI initial). (**C**) The memory index is calculated by dividing CI test by the mean of the sham control courtship levels (CI sham).

To relate escape climbing performance and courtship (performance, learning, and short-term memory) to each other, the same flies were also tested in the courtship conditioning assay (Fig 4A). However, courtship motor behavior requires animals that can walk. Therefore, aged but locomotory impaired flies were excluded from the courtship analysis and thus from the comparison at that day. The selection was taking place daily in the startle assay. Flies failing to climb to the top of the vial (non-climbers) did not further participate in courtship performance analysis but their physiological status was examined daily until death. Non-climbers exhibited moderate, severe or complete climbing disability alone, or together with a deficit in leg mobility and responsiveness. During the courtship assay, male climbers were presented for 1h to mated females (trainer female) and courtship index was measured during the first 10 minutes (CI initial, Fig 4A, black bars) and the last 10 minutes (CI final, Fig 4A, gray bars). The ratio of CI final/CI initial is the learning index during training, which shows no statistical differences between 4 and 10 weeks of age (Fig 4B). Next, 5 minutes after the 1h training, the trained male was presented to a young virgin female (CI test, Fig 4A, dark gray bars). In parallel, same aged, non-trained males were presented to a young virgin female (CI Sham, Fig 4A, white bars). The ratio of CI test divided by CI Sham yields the 5 minutes short term memory (STM) index, which also shows no statistically significant differences between 4 and 10 weeks of age (Fig 4C).

Courtship motor performance is robust during all ages, CI initial and CI Sham are constantly high (>0.9) between 4 and 8 weeks, but decay after the 8th week of age (Fig 4A). Further analysis of courtship (orientation), singing, licking and attempting copulation latencies (the time between the introduction of flies in the courtship chamber to the time of the first exhibition of any of the particular behaviors) reveal a gradual age-dependent increase in the latency of male engagement towards all three female partners (S1 Fig, Table 1). However, at any given age tested no differences are found in the latencies of engagement with the mated trained, the virgin tester, or the virgin control females (S1 Fig, and see below). The age-related latency increase accumulates to a decrease of CI in 10 weeks old flies (Fig 4A). Learning index during training varies between 0.45 to 0.70 with no significant differences between age groups and no clear trend over time (Fig 4B), indicating persistence of learning ability throughout ages. The STM memory index varies between 0.7 to 0.85 (Fig 4C) without statistically significant differences between ages. Therefore, in animals without major physical impairments both courtship learning and STM are stable throughout life. In contrast, climbing and courtship motor performance is robust in mid life up to the age of 8 weeks but declines at old ages (8–10 weeks), although 10 weeks old, wellderly flies can still perform.

**Table 1:** Comparison of latencies for male courtship parameters depicted in Fig 4B. p -values from One-way ANOVA, Kruskal-Wallis test, Tukey’s multiple comparisons test. Values in bold indicate significance.

	Courtship Latency	Singing Latency	Licking Latency	Att. Cop. Latency
4W Tester vs. 4W Trainer	>0.9999	>0.9999	>0.9999	0.956
4W Tester vs. 4W Sham	>0.9999	>0.9999	>0.9999	>0.9999
4W Trainer vs. 4W Sham	>0.9999	>0.9999	>0.9999	>0.9999
6W Tester vs. 6W Trainer	>0.9999	>0.9999	>0.9999	0.9934
6W Tester vs. 6W Sham	>0.9999	>0.9999	>0.9999	0.0031
6W Trainer vs. 6W Sham	>0.9999	>0.9999	>0.9999	<**0.0001**
8W Tester vs. 8W Trainer	>0.9999	>0.9999	>0.9999	0.9999
8W Tester vs. 8W Sham	0.4614	>0.9999	>0.9999	0.2567
8W Trainer vs. 8W Sham	0.0039	0.0231	<0.0001	0.5728
10W Tester vs. 10W Trainer	>0.9999	>0.9999	>0.9999	0.9539
10W Tester vs.10W Sham	>0.9999	>0.9999	>0.9999	0.7747
10W Trainer vs. 10W Sham	>0.9999	>0.9999	>0.9999	>0.9999
4W Tester vs. 6W Tester	0.468	0.3145	**0.0011**	**0.0026**
4W Tester vs. 8W Tester	0.121	**0.0433**	**<0.0001**	**0.0001**
4W Tester vs. 10W Tester	0.0591	0.1366	**<0.0001**	**0.0355**
6W Tester vs. 8W Tester	>0.9999	>0.9999	>0.9999	>0.9999
6W Tester vs. 10W Tester	>0.9999	>0.9999	0.2871	>0.9999
8W Tester vs. 10W Tester	>0.9999	>0.9999	>0.9999	>0.9999
4W Trainer vs. 6W Trainer	0.7131	**<0.0001**	**0.0524**	**0.0013**
4W Trainer vs. 8W Trainer	**0.0015**	**0.001**	**<0.0001**	**<0.0001**
4W Trainer vs. 10W Trainer	**0.0134**	**0.0003**	**<0.0001**	**<0.0001**
6W Trainer vs. 8W Trainer	0.2115	**0.0518**	**0.0005**	**0.0098**
6W Trainer vs. 10W Trainer	0.4979	>0.9999	**<0.0001**	**0.0846**
8W Trainer vs. 10W Trainer	>0.9999	>0.9999	**0.025**	>0.9999
4W Sham vs. 6W Sham	0.4722	0.2239	**0.0155**	**<0.0001**
4W Sham vs. 8W Sham	>0.9999	0.0002	**<0.0001**	**0.0004**
4W Sham vs. 10W Sham	0.0131	0.0003	**<0.0001**	**<0.0001**
6W Sham vs. 8W Sham	>0.9999	0.2272	0.1476	>0.9999
6W Sham vs. 10W Sham	0.8516	0.0998	0.0739	>0.9999
8W Sham vs. 10W Sham	0.2314	>0.9999	>0.9999	>0.9999

**Fig 5:**
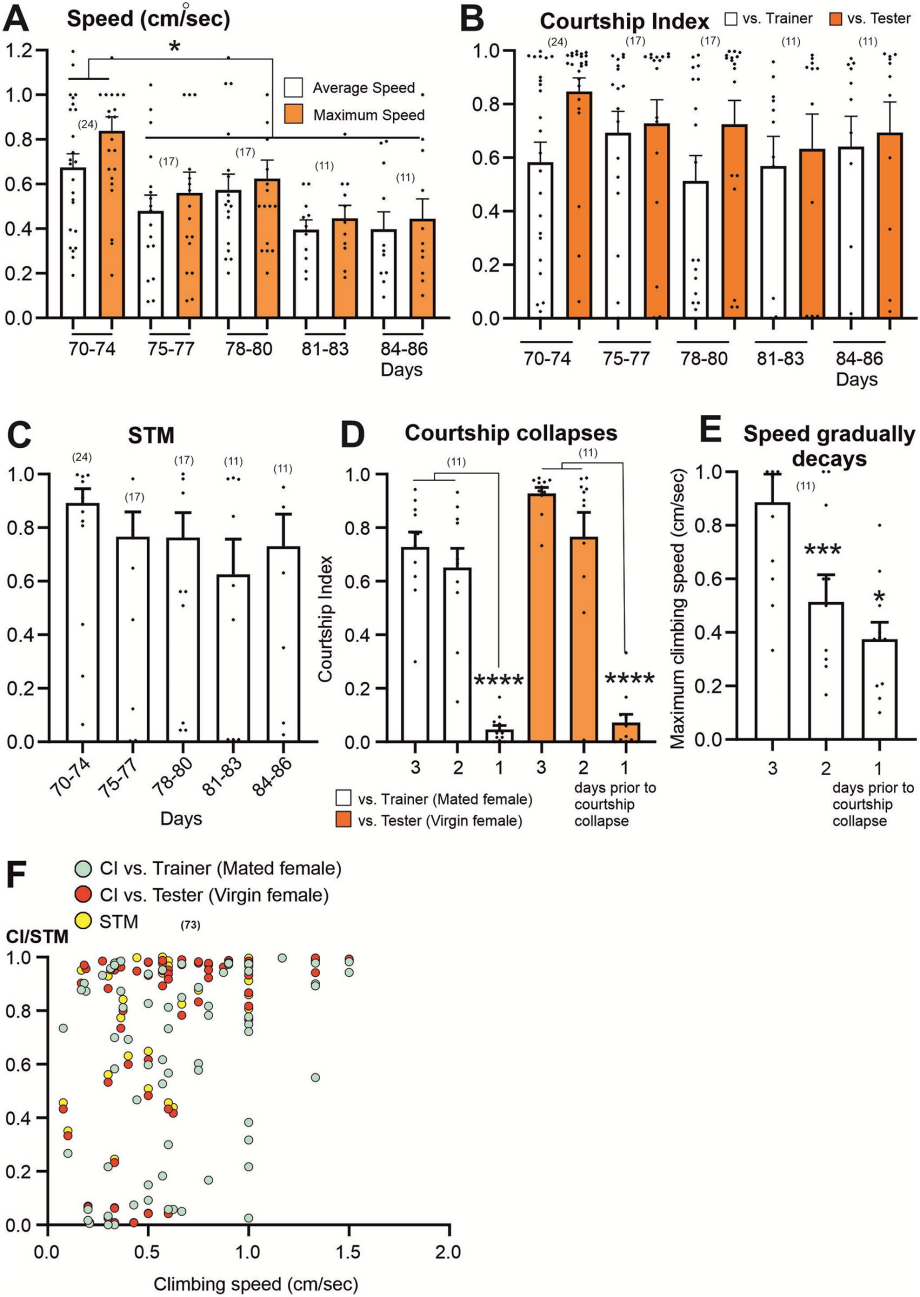
In old flies, speed decays gradually whereas courtship performances collapses, but a weak-to-moderate correlation is found between speed and STM. **(A)** Climbing speed measurements during the last days of the cohort (>10 weeks). Not the final drop of speed past the 75^th^ day. **(B)** Courtship and Short Term Memory (STM) Indexes remain stable during the last days. (**C**) Patterns of change in courtship and climbing speed during the last three days prior to courtship collapse. Note that courtship against the tester (CI test) and the trainer (CI initial) abruptly drop to very low levels whereas the climbing speed declines gradually. (One-Way ANOVA, Kruskal-Wallis multiple comparison test, * p<0.05, ** p<0.01, *** p<0.001, **** p<0.0001). **(D)** No correlation between speed and both courtship (CI trainer, CI tester) and STM indexes. Note that a slow individual can out-perform in courtship engagement and vice versa.

To further define the patterns of decay in climbing speed and courtship performance in highly advanced ages, we conducted daily assays in twenty-five old (>10 weeks) wellderly males until they developed pre-death impairments or suddenly died (Fig 3A, Fly # 1–25). The final decrease of climbing speed of elderly males occurs past the 75^th^ day of the cohort’s age (Fig 5A) but CI and STM do not change during this time (Fig 5B). Latencies in the initiation of courtship rituals remain stable throughout this period as well, with shorter latencies in courtship engagements towards the tester (virgin female; S2 Fig). Independent of the age at death, high levels of courtship performance against virgin and mated female partners drop suddenly, within one day (Fig 5C) to negligible levels (CI<0.1). Therefore, on average courtship collapses occurs2.5 days prior to impairment onset and ~4 days prior to death (Fig 3F). By contrast, climbing speed declines gradually during the time when courtship performance collapses in the same individuals (Fig 5C). One possibility to explain how gradual decreases in climbing speed through late age could cause a sudden collapse of courtship within one day would be a threshold for climbing/walking speed below which animals become incapable to court. However, this is not the case. First, plotting climbing speed versus CI, STM and the latency in the initiation of each courtship sub-behavior against the trainer (mated female) and tester (virgin female) for each individual male reveals either no correlation (ages: 4 weeks-10; Table 2) or week correlation (age: >10 weeks; Table 3). Second, plotting speed versus CI for old flies shows that some of the slowest males acquired high scores in in courtship performance and *vice versa*, other flies showed low CI but still had medium walking/climbing speed (Fig 5D) These data indicate that the sudden drop in courtship performance at late ages is mechanistically independent of the gradual age-related decline in locomotion speed.

**Table 2:** 4–10 week old males. R and p values from Spearman rank correlation test. Values in bold indicate significance.

Age	Speed vs. CI initial	Speed vs. CI test/STM	Speed vs. Learning Index
4 weeks	r = -0.09049 p = 0.5193	r = -0.06935 p = 0.6287	r = -0.3567 p = 0.0204
6 weeks	r = 0.0266 p = 0.869	r = 0.2611 p = 0.070	r = 0.2333 p = 0.147
8 weeks	r = 0.1523 p = 0.361	r = 0.1089 p = 0.492	r = -0.0126 p = 0.942
10 weeks	**r = 0.5042** **p = 0.019**	r = 0.3515 p = 0.129	r = -0.01355 p = 0.955

**Table 3:** >10 week old males. R and p values from Spearman rank correlation test. Values in bold indicate significance.

	**Towards trainer (Initial 10 mins)**				
Age:70–85 d	Speed vs. Orientation Latency	Speed vs. Singing Latency	Speed vs. Licking Latency	Speed vs. At. Copulation Latency	Speed vs. CI Initial
**r**	-0,1059	-0,2100	-0,2318	-0,1805	0,3059
**P**	0,3831	0,1238	0,1129	0,2353	**0,0085**
	**Towards tester (Initial 10 mins)**				
Age:70–85 d	Speed vs. Orient. Latency	Speed vs. Singing Latency	Speed vs. Licking Latency	Speed vs. At. Copulation Latency	Speed vs. CI test./ STM
**r**	-0,4022	-0,4171	-0,3212	-0,3322	0,5169
**P**	**0,0004**	**0,0005**	**0,0202**	**0,0184**	**<0,0001**

Importantly, the orientation of the male towards the female, which is the first step in the escalating sequence of courtship rituals (measured as courtship/orientation latency, S1 and S2 Figs), is largely independent of climbing performance across ages, including very old wellderly flies (Tables 2 and 3) and even impaired individuals (our observation). This suggests that both courtship target identification by the male and the innate interest to court are abilities that persists until almost death. Overall, the data indicate that during biological aging of otherwise healthy flies, age-related cognitive and motor decline occur with different dynamics and largely independently from each other (see discussion).

## Discussion

We used two robust innate behaviors with high evolutionary relevance for cross-sectional and longitudinal assessment of aging-related cognitive and motor decline. Adequate courtship behavior ensures reproductive success and escape climbing is important for survival. Below we will first discuss the impact of aging on escape climbing, second age-related changes in courtship performance as well as in courtship learning and memory, and third, the relationship between cognitive and motor decline.

### The patterns of age-related escape climbing speed decrease

Startle induced escape responses can be elicited until few hours prior to death, unless flies suffer from locomotion impairments that make the task impossible. Such severely impaired flies were excluded from the analysis because we aimed at identifying the patterns of healthy motor aging. It is known that locomotion speed declines with age across species, including humans [35, 36] and *Drosophila* [19]. Thus, age-related reductions in locomotion speed are a conserved feature of aging from flies to humans. However, both our cross-sectional as well as our longitudinal analyses show that once flies reach mid age (4 weeks), locomotion speed remains stable until the age of 8 weeks, but thereafter declines gradually again until late life (8–11 weeks). The same is the case for the responsiveness to the startle stimulus. Therefore, mid life is characterized by a locomotion performance plateau. This mid age locomotor performance plateau in *Drosophila* is not in agreement with a previous report [19], but there, the Oregon-R wildtype strain used in our analysis has been tested only until 6 weeks of age, which might have hidden the performance plateau from the analysis. Moreover, when assessing climbing ability by measuring speed during the during the first 4 seconds of climbing [19, 37], or as percentage of flies at the top vial [38, 39], a gradual reduction in negative geotaxis has been found already during mid ages (4–8 weeks). By contrast, we did not find significant differences in climbing speed during mid ages. This discrepancy might be due to assay-type differences as our assay measures the time to complete climbing a distance of 6 cm on a vertical wall (equaling average speed).

Similar to our findings in *Drosophila* a plateau of locomotion performance has been observed in human subjects. Although human physical performance often declines during mid age, assessment of marathon and half-marathon runners aged 20 to 79 years revealed no physical performance decline until the age of 55 years in subjects with healthy life-style. Parallel assessment with life-style questionaries has yielded the conclusion that human mid age physical performance decline is mainly attributed to life-style but not to biological aging [40]. In our analysis on *Drosophila*, lifestyle was not a factor, because all animals were raised under identical laboratory conditions. As mentioned above, during late life, climbing speed and thus locomotor performance declines gradually both in *Drosophila* (8–11 weeks/56–77 days, this study) as well as in humans (55 to 79 years; [40].

As mentioned above, the ability to respond with escape climbing in the startle assay remains through old ages, but the duration to complete the task is significantly increased in very old male flies (10 weeks). In addition, the response magnitude scales with stimulus intensity, at least for moderate as compared to strong stimuli. This scalability of the response persists to old ages (even in 10 weeks old males). Taken together, these data suggest sufficient preservation of sensory-motor escape circuit functional integrity to complete the startle task, though with decreased performance speed at very old ages. By contrast, the integrator that translates input amplitude into response magnitude seems resilient through all ages measured.

### The patterns of courtship motor performance aging

Courtship behavior comprises a complex sequence of different motor tasks (orientation towards the female, “love-song” production by wing movement, chasing, licking, attempting copulation) which are orchestrated by communication between female and male flies [21, 41]. The courtship index (CI) resembles the percentage of time during which any of these motor tasks is executed and was assessed during 10 minutes time periods in this study. Therefore, in contrast to escape climbing, which is a single motor task that is completed within seconds, CI rather represents a measure for perseverance of a complex motor behavior. Our longitudinal analysis of CI, and thus courtship motor performance, revealed a similar pattern of age- related decline as escape climbing speed. CI remains stable during mid life (four to eight weeks of age) but then declines significantly in old flies (>10 weeks). A similar tendency is apparent in the cross-sectional analysis, but this is not statistically significant, underscoring the importance of longitudinal assessment of individuals. These data indicate that both locomotor speed and motor behavioral perseverance are stable during mid life but start declining at similar old ages. Interestingly, the latencies to the first execution of the different parts of the courtship behavioral sequence remain unaltered throughout mid and late life, indicating that the initial interest of male flies to court remains unaltered, even in the face of declining motor abilities. In fact, even physically impaired males that are during their last two days of life, cannot sing and barely walk, show immediate orientation toward a virgin female, underscoring the robustness of innate male reproductive interest and target identification against biological aging.

During late life climbing speed decreases gradually, whereas courtship behavior collapses within one day. An obvious interpretation is that the gradual locomotion speed decline in old flies causes the courtship sequence to be interrupted, because the male can simply not chase the young, virgin female anymore. However, although there is a low-to-moderate correlation between walking speed and CI in old flies (>10 weeks), some of the slowest flies still show high CIs, and some reasonably fast flies show CIs close to zero. Therefore, the sudden collapse of courtship performance in old flies is unlikely a consequence of slow walking speed. In fact, old males that exhibit courtship performance collapse can still locomote, detect the female, and initiate motor behavior because they all have passed the startle assay, and they all orient toward the female (and occasionally even start following her). Therefore, courtship performance collapse is unlikely due to failure of the motor or sensory parts of the circuit. Instead, it seems more likely that it is caused by defects in central circuitry for decision making or for maintaining a motor behavioral sequence. However, the dissection of the underlying molecular and cellular mechanisms requires spatially and temporally controlled genetic manipulation is beyond the scope of this study.

### The patterns of courtship learning and memory aging

Although courtship is an innate behavior, its execution can be modified by learning. If courting males are rejected by a mated female, they subsequently engage less into courtship behavior when presented with a virgin female [42]. In young male flies this memory can be retrieved either as STM shortly after the training with a mated female (here after 5 minutes) or as LTM 24 h after training [43]. Knowledge on age-related changes of courtship memory is sparse, but it has been shown that courtship learning and memory assessed at 1h after training exists in mid aged flies (45 day of age), though reduced as compared to young animals (5 days of age,). We assessed courtship learning and memory in parallel with motor aging during mid and late life of *Drosophila*. We show that aging differentially affects different forms of courtship memory. Learning and courtship STM persist into late life while courtship LTM as assessed 24hrs after training is absent already in mid aged flies. Similarly, specific olfactory memories are significantly reduced or absent in mid aged flies. Aversive olfactory memory acquired in a single olfactory conditioning session comprises three phases: short-term memory (STM), midterm memory (MTM), and longer-lasting anesthesia-resistant memory (ARM). Aging-related olfactory memory loss has been attributed to the decline of the amnesiac-dependent MTM [27, 28, 44, 45], but LTM is also impaired [28, 30, 46]. Similar to the loss of olfactory MTM/LTM we find that courtship LTM is lost in mid aged flies. Given that male flies do not court constantly during the hours of conditioning [33], LTM is probably formed by spaced training in a single training session. Similarly, spaced-training induced olfactory LTM is absent in old ages. Olfactory LTM depends on protein synthesis and the activity of cAMP response element-binding protein (Creb) during conditioning [47–50]. Aging impairs the activation of CSW-dependent mitogen-activated protein kinase MAPK; [51] and Raf/MAPK pathway during conditioning [52, 53]. By contrast, in olfactory learning, STM, and protein-synthesis-independent/anesthesia-resistant long-term memory LTM/ARM [27, 28, 30, 46] are more resilient to aging. Although it remains speculative whether similar mechanisms are at act during courtship memory, we have shown that courtship learning and STM persist into ages when LTM is absent, like it is the case for different olfactory memories. An attractive hypothesis is that memories dependent on protein synthesis are less resilient age to related mis-regulation of proteostasis. However, other LTMs, such as a body size memory that is formed during a critical period in young adults remains stable through all ages tested [54]. Therefore, in *Drosophila* different types of LTM can show different patterns of age-related decline.

The absence of courtship LTM already after four weeks of adult life enabled us to conduct longitudinal assessment of learning and STM in the courtship assay during mid and late life, because we could exclude the interference of LTM with the repeated measurements throughout life from the same individuals.

In contrast to courtship LTM, learning during courtship as well as STM remain unaffected in late life until courtship behavior collapses entirely. This seems biologically relevant, because not learning that an ongoing or just waved courtship attempt is or has been futile would cause useless investment into a goal that cannot be achieved. In fact, recent data on olfactory memory indicate that the formation or retention of appetitive memory with survival benefits is maintained in aged flies [28]. These data indicate that mechanisms that protect against cognitive loss may evolve predominantly for memories that are biologically meaningful also at late ages.

### Motor and cognitive decline show only moderate correlations and only during late life

Based on reported positive correlations between specific aspects of mobility and cognitive decline in humans [9–12] during aging, we originally hypothesized that age-related decreases in climbing speed may predict decline of courtship learning and memory defects. Our data largely reject this hypothesis. First, although climbing speed is slower in mid aged flies than in young flies, this cannot predict cognitive decline. Second, during mid life (4 to 8 weeks of age) we found no period of time during which motor decline can predict any aspect of cognitive decline in courtship learning and memory, or *vice versa*. LTM is already gone and STM remains unaltered through mid life. Only during late life (flies > 10 weeks) there is a moderate correlation between climbing speed and STM, but not between climbing speed and learning. Therefore, a common underlying cause for the differential age-related decline of different motor and cognitive behaviors seems unlikely but cannot be excluded. An alternative hypothesis is one shared common cause but different thresholds for the decline of different behavioral performances.

In our view the data favor the idea that various aspects of motor and cognitive decline are affected differentially by the aging process. This is in line with differential vulnerabilities of different types of neurons and neural circuits [13, 14], as reported across species. A long-term goal is thus to connect the differential decline of learning and memory versus locomotion to neural circuitry and/or differential aging of neurons of different neurotransmitter classes. In the Drosophila olfactory system, aging associated circuit degeneration has been linked to a single class of cholinergic projection neurons [13]. Although a rigorous analysis of this question for the behaviors analyzed in this study requires genetic manipulation, it is known that the Drosophila brain does not display major neurodegeneration or apoptosis with increasing age. By contrast, subtle changes in the ultrastructure of central neurons [55], motoneuron axonal terminal morphology [56, 57], and at the level of neuromodulators [39] have been associated to aging. In particular, the degeneration of specific dopaminergic neuron clusters has been associated with an accelerated decline of startle-induced negative geotaxis in aging flies [58, 59]. Although dopamine modulates a wide array of Drosophila behaviors [60], including locomotion, courtship, and learning and memory, different dopaminergic neurons are involved in the different behaviors. Therefore, a potential future avenue could be to test whether differential behavioral decline is associated with differential functional decline of different populations of dopaminergic neurons. By contrast, other aspects of behavioral decline are associated with other transmitter classes. For example, long term courtship memory (LTM) declines earlier, and LTM formation requires glial-dependent inhibition of glutamate signaling during memory consolidation. It has been reported that aging disrupts this process by inhibiting the Klg-Repo-EAAT1 pathway [61]. Moreover, age-dependent decreases in the expression or function of mGluR impairs sleep and memory in flies [62]. However, a causal link between differential decline of different behaviors during aging and transmitter class and/or neuron type requires additional experiments with precise genetic manipulation.

In *Drosophila*, genes and neural circuits underling escape motor responses [63] and courtship [64] are well characterized. Combining the recent success in circuit mapping in the relatively simple *Drosophila* brain, at least when compared to humans, with the versatile tools for genetic manipulation with exquisite spatial and temporal resolution makes *Drosophila* a useful genetic model to probe the molecular and cellular mechanisms underlying differential decline of distinct aspects of motor and cognitive function during biological aging.

## Supporting information

S1 Fig Courtship rituals latencies, that is the time it takes for an individual to initiate any of the behavior, increase in late life. (JPG)

S2 Fig Latencies in the initiation of courtship rituals remain stable in old flies (>10 weeks). (JPG)

## References

[1] Christensen K, Doblhammer G, Rau R, VaupeI JW. Ageing populations: the challenges ahead. Lancet 2009;374: 1196–1208. doi: 10.1016/S0140-6736(09)61460-4 19801098 PMC2810516

[2] Brown GC. Living too long: the current focus of medical research on increasing the quantity, rather than the quality, of life is damaging our health and harming the economy. EMBO Rep 2015;16: 137–141. doi: 10.15252/embr.201439518 25525070 PMC4328740

[3] Kenyon CJ. The genetics of ageing. Nature 2010;464(7288): 504–12. doi: 10.1038/nature08980 20336132

[4] Gaitanidis A, Dimitriadou A, Dowse H, Sanyal S, Duch C, Consoulas C. Longitudinal assessment of health-span and pre-death morbidity in wild type *Drosophila*. Aging (Albany NY) 2019;27,11(6): 1850–1873. doi: 10.18632/aging.101880 30923256 PMC6461171

[5] Kauwe JSK, Goate A. Genes for a “Wellderly” Life. Trends Mol Med 2016;22(8): 637–639. doi: 10.1016/j.molmed.2016.05.011 27312143 PMC5499691

[6] Fontana L, Partridge L. Promoting health and longevity through diet: from model organisms to humans. Cell 2015;161: 106–118. doi: 10.1016/j.cell.2015.02.020 25815989 PMC4547605

[7] López-Otín C, Blasco MA, Partridge L, Serrano M, Kroemer G. The hallmarks of aging. Cell 2013;6153(6): 1194–1217. doi: 10.1016/j.cell.2013.05.039 23746838 PMC3836174

[8] Christensen H, Mackinnon AJ, Korten A, Jorm AF. The “common cause hypothesis” of cognitive aging: Evidence for not only a common factor but also specific associations of age with vision and grip strength in a cross-sectional analysis. Psychology and Aging 2001;16(4): 588–599. doi: 10.1037//0882-7974.16.4.588 11766914

[9] Clouston SA, Brewster P, Kuh D, Richards M, Cooper R, et al. The dynamic relationship between physical function and cognition in longitudinal aging cohorts. Epidemiol Rev 2013;35(1): 33–50. doi: 10.1093/epirev/mxs004 23349427 PMC3578448

[10] Demnitz N, Esser P, Dawes H, Valkanova V, Johansen-Berg H, et al. A systematic review and meta-analysis of cross-sectional studies examining the relationship between mobility and cognition in healthy older adults. Gait Posture 2016;50: 164–174. doi: 10.1016/j.gaitpost.2016.08.028 27621086 PMC5081060

[11] Morris R, Lord S, Bunce J, Burn D, Rochester L. Gait and cognition: Mapping the global and discrete relationships in ageing and neurodegenerative disease. Neurosci Biobehav Rev. 2016;64: 326–345. doi: 10.1016/j.neubiorev.2016.02.012 26915926

[12] Tian Q, An Y, Resnick SM, Studenski S. The relative temporal sequence of decline in mobility and cognition among initially unimpaired older adults: Results from the Baltimore Longitudinal Study of Aging. Age Ageing 2017;46(3): 445–451. doi: 10.1093/ageing/afw185 27744302 PMC5860013

[13] Hussain A, Pooryasin A, Zhang M, Loschek LF, La Fortezza M, Friedrich AB, et al. Inhibition of oxidative stress in cholinergic projection neurons fully rescues aging-associated olfactory circuit degeneration in *Drosophila*. eLife 2018; 7:e32018. doi: 10.7554/eLife.32018 29345616 PMC5790380

[14] Mattson MP, Magnus T. Ageing and neuronal vulnerability. Nat Rev Neurosci. 2006;7(4): 278–294. doi: 10.1038/nrn1886 16552414 PMC3710114

[15] Fu H, Hardy J, Duff KE. Selective vulnerability in neurodegenerative diseases. Nat Neurosci. 2018;21: 1350–1358. doi: 10.1038/s41593-018-0221-2 30250262 PMC6360529

[16] Dumitriu D, Hao J, Hara Y. Selective changes in thin spine density and morphology in monkey prefrontal cortex correlate with aging-related cognitive impairment. J Neurosci. 2010;30(22): 7507–7517. doi: 10.1523/JNEUROSCI.6410-09.2010 20519525 PMC2892969

[17] Lester WA, Moffat DS, Wiener MJ, Barnes AC, Wolbers T. The Aging Navigational System. Neuron 2017;95(5): 1019–1035. doi: 10.1016/j.neuron.2017.06.037 28858613 PMC5659315

[18] Middleton A, Fritz LS, Lusardi M. Walking speed: the functional vital sign. J Aging Phys Act 2015;23(2): 314–322. doi: 10.1123/japa.2013-0236 24812254 PMC4254896

[19] Rhodenizer D, Martin I, Bhandari P, Pletcher SD, Grotewiel M. Genetic and environmental factors impact age-related impairment of negative geotaxis in *Drosophila* by altering age-dependent climbing speed. Exp Gerontol. 2008;43: 739–748. doi: 10.1016/j.exger.2008.04.011 18515028 PMC2591094

[20] Stamper BLN, Ypser JR, Kechris K, Kitzenberg DA, Tedesco PM, Johnson TE. Movement decline across lifespan of *Caenorhabditis elegans* mutants in the insulin/insulin-like signaling pathway. Aging Cell 2018;17(1): e12704. doi: 10.1111/acel.12704 29214707 PMC5770877

[21] Griffith LC, Ejima A. Courtship learning in *Drosophila melanogaster*: diverse plasticity of a reproductive behavior. Learn Mem 2009;16(12): 743–750. doi: 10.1101/lm.956309 19926779 PMC4419844

[22] Grotewiel MS, Martin I, Bhandari P, Cook-Wiens E. Functional senescence in *Drosophila melanogaster*. Ageing Res Rev. 2005;4(3): 372–397. doi: 10.1016/j.arr.2005.04.001 16024299

[23] Blumenthal HT. The aging-disease dichotomy: true or false? J Gerontol A Biol Sci Med Sci. 2003;58(2): 138–145. doi: 10.1093/gerona/58.2.m138 12586851

[24] Howlett SE. Assessment of Frailty in Animal Models. Interdiscip Top Gerontol Geriatr 2015;41: 15–25. doi: 10.1159/000381131 26301976

[25] Antoch MP, Wrobel M, Kuropatwinski KK, Gitlin I, Leonova KI, et al. Physiological frailty index (PFI): quantitative in-life estimate of individual biological age in mice. Aging 2017;9(3): 615–626. doi: 10.18632/aging.101206 28325885 PMC5391222

[26] Gill TM, Gahbauer EA, Han L, Allore HG. Trajectories of disability in the last year of life. N Engl J Med 2010;362: 1173–1180. doi: 10.1056/NEJMoa0909087 20357280 PMC2877372

[27] Tamura T, Chiang AS, Ito N, Liu HP, Horiuchi J, et al,. Aging specifically impairs amnesiac-dependent memory in *Drosophila*. Neuron. 2003;40(5): 1003–1011. doi: 10.1016/s0896-6273(03)00732-3 14659098

[28] Tonoki A, Davis RL. Aging impairs protein-synthesis-dependent long-term memory in *Drosophila*. J Neurosci. 2015;35(3): 1173–1180. doi: 10.1523/JNEUROSCI.0978-14.2015 25609631 PMC4300323

[29] Gupta GV, Scheunemann L, Eisenberg T, Mertel S, Bhukel A, et al. Restoring polyamines protects from age-induced memory impairment in an autophagy-dependent manner. Nat Neurosci. 2013;16:1453–1460. 16:1453–60. doi: 10.1038/nn.3512 23995066

[30] Haddadi M, Jahromi RS, Sagar BC, Patil KR, Shivanandappa T, et al. Brain aging, memory impairment and oxidative stress: A study in *Drosophila melanogaster*. Behavioural Brain Res. 2014;259: 60–69. doi: 10.1016/j.bbr.2013.10.036 24183945

[31] Tonoki A, Ogasawara M, Yu Z, Itoh M. Appetitive Memory with Survival Benefit Is Robust Across Aging in *Drosophila*. J Neurosci. 2020;40 (11): 2296–2304. doi: 10.1523/JNEUROSCI.2045-19.2020 31992587 PMC7083296

[32] Ejima A, Griffith LC. Measurement of Courtship Behavior in *Drosophila melanogaster*. CSH Protoc. 2007;pdb.prot4847. doi: 10.1101/pdb.prot4847 21356948

[33] Koemans TS, Oppitz C, Donders R, van Bokhoven H, Schenck A, et al. M. Drosophila Courtship Conditioning as a Measure of Learning and Memory. J Vis Exp. 2017; (124):55808. doi: 10.3791/55808 28605393 PMC5608251

[34] Sakai T, Tamura T, Kitamoto T, Kidokoro Y. A clock gene, period, plays a key role in long-term memory formation in *Drosophila*. Proc Natl Acad Sci USA. 2004;101: 16058–16063. doi: 10.1073/pnas.0401472101 15522971 PMC528738

[35] McGibbon CA, Krebs DE. Age-related changes in lower trunk coordination and energy transfer during gait. J Neurophysiol. 2001;85(5): 1923–1931. doi: 10.1152/jn.2001.85.5.1923 11353009

[36] Samson MM, Crowe A, de Vreede PL, Dessens JA, Duursma SA, Verhaar HJ. Differences in gait parameters at a preferred walking speed in healthy subjects due to age, height and body weight. Aging (Milano). 2001;13(1): 16–21. doi: 10.1007/BF03351489 11292147

[37] Gargano JW, Martin I, Bhandari P, Grotewiel MS. Rapid iterative negative geotaxis (RING): a new method for assessing age-related locomotor decline in Drosophila. Exp Gerontol. 2005;40(5):386–95. doi: 10.1016/j.exger.2005.02.005 15919590

[38] Simon AF, Liang DT, Krantz DE. Differential decline in behavioral performance of *Drosophila melanogaster* with age. Mech Ageing Dev. 2006;127(7): 647–651. doi: 10.1016/j.mad.2006.02.006 16616306

[39] Liao S, Broughton S, Nässel DR. Behavioral Senescence and Aging-Related Changes in Motor Neurons and Brain Neuromodulator Levels Are Ameliorated by Lifespan-Extending Reproductive Dormancy in Drosophila. Front Cell Neurosci. 2017; 11:111. doi: 10.3389/fncel.2017.00111 28503133 PMC5408790

[40] Leyk D, Rüther T, Wunderlich M, Sievert A, Essfeld D, et al,. Physical performance in middle age and old age: good news for our sedentary and aging society. Dtsch Arztebl Int. 2010;107(46): 809–816. doi: 10.3238/arztebl.2010.0809 21151416 PMC2999945

[41] Hall JC. The Mating of a Fly. Science 1994;264: 1702–1714. doi: 10.1126/science.8209251 8209251

[42] Siegel W.R, Hall C.J. Conditioned responses in courtship behavior of normal and mutant *Drosophila*. Proc Natl Acad Sci U S A. 1979;76 (7): 3430–3434. doi: 10.1073/pnas.76.7.3430 16592682 PMC383839

[43] McBride SM, Giuliani G, Choi C, Krause P, Correale D, et al. Mushroom body ablation impairs short-term memory and long-term memory of courtship conditioning in *Drosophila melanogaster*. Neuron. 1999;24(4): 967–77. doi: 10.1016/s0896-6273(00)81043-0 10624959

[44] Sean M.J, McBride C.H.C.; Schoenfeld B.P, Bell A.J, Liebelt D.A et al. Pharmacological and Genetic Reversal of Age-Dependent Cognitive Deficits Attributable to Decreased presenilin Function. J Neurosci. 2010;30(28): 9510–9522. doi: 10.1523/JNEUROSCI.1017-10.2010 20631179 PMC2917645

[45] Saitoe M, Horiuchi J, Tamura T, Ito N. *Drosophila* as a novel animal model for studying the genetics of age-related memory impairment. Rev Neurosci. 2005;16(2): 137–149. doi: 10.1515/revneuro.2005.16.2.137 15957577

[46] Mery F. Aging and its differential effects on consolidated memory forms in Drosophila. Exp Gerontol. 2007;42(1–2):99–101. doi: 10.1016/j.exger.2006.06.004 16860960

[47] Tully T, Cambiazo V, Kruse L. The role of cAMP response element-binding protein in Drosophila long-term memory. J Neurosci. 2004;24(40):8823–8. doi: 10.1523/JNEUROSCI.4542-03.2004 15470148 PMC6729945

[48] Yin JCP, Del Vecchio M, Zhou H, Yin T. CREB as a Memory Modulator: induced expression of a dCREB2 activator isoform enhances long-term memory in *Drosophila*. Cell. 1995; 81(1):107–115. doi: 10.1016/0092-8674(95)90375-5 7720066

[49] Perazzona B, Isabel G, Preat T, Davis RL. The role of cAMP response element-binding protein in Drosophila long-term memory. J Neurosci. 2004;24(40): 8823–8. doi: 10.1523/JNEUROSCI.4542-03.2004 15470148 PMC6729945

[50] Yu D, Akalal DB, Davis RL. Drosophila alpha/beta mushroom body neurons form a branch-specific, long-term cellular memory trace after spaced olfactory conditioning. Neuron. 2006;52(5): 845–55. doi: 10.1016/j.neuron.2006.10.030 17145505 PMC1779901

[51] Pagani MR, Oishi K, Gelb BD, Zhong Y. The phosphatase SHP2 regulates the spacing effect for long-term memory induction. Cell. 2009;139(1): 186–98. doi: 10.1016/j.cell.2009.08.033 19804763 PMC2770243

[52] Zhang X, Li Q, Wang L, Liu ZJ, Zhong Y. Active Protection: Learning-Activated Raf/MAPK Activity Protects Labile Memory from Rac1-Independent Forgetting. Neuron. 2018;98(1):142–155.e4. doi: 10.1016/j.neuron.2018.02.025 29551489

[53] Mo H, Wang L, Chen Y, Zhang X, Huang N, Liu T, et al. Age-related memory vulnerability to interfering stimuli is caused by gradual loss of MAPK-dependent protection in Drosophila. Aging Cell. 2022 Jun;21(6):e13628. doi: 10.1111/acel.13628 35570367 PMC9197400

[54] Krause T, Spindler L, Poeck B, Strauss R. *Drosophila* Acquires a Long-Lasting Body-Size Memory from Visual Feedback. Curr Biol. 2019;29(11): 1833–1841. doi: 10.1016/j.cub.2019.04.037 31104933

[55] Martín-Peña A, Acebes A, Rodríguez JR, Sorribes A, de Polavieja GG, Fernández-Fúnez P, et al. Age-independent synaptogenesis by phosphoinositide 3 kinase. J Neurosci. 2006;26(40):10199–208. doi: 10.1523/JNEUROSCI.1223-06.2006 17021175 PMC6674615

[56] Beramendi A, Peron S, Casanova G, Reggiani C, Cantera R. Neuromuscular junction in abdominal muscles of Drosophila melanogaster during adulthood and aging. J Comp Neurol. 2007;501(4): 498–508. doi: 10.1002/cne.21253 17278125

[57] Wagner N, Laugks U, Heckmann M, Asan E, Neuser K. Aging *Drosophila melanogaster* display altered pre- and postsynaptic ultrastructure at adult neuromuscular junctions. J Comp Neurol. 2015;523(16): 2457–75. doi: 10.1002/cne.23798 25940748

[58] Riemensperger T, Issa AR, Pech U, Coulom H, Nguyễn MV, Cassar M, et al. A single dopamine pathway underlies progressive locomotor deficits in a Drosophila model of Parkinson disease. Cell Rep. 2013;5(4): 952–60. doi: 10.1016/j.celrep.2013.10.032 24239353

[59] Vaccaro A, Issa AR, Seugnet L, Birman S, Klarsfeld A. Drosophila Clock Is Required in Brain Pacemaker Neurons to Prevent Premature Locomotor Aging Independently of Its Circadian Function. PLoS Genet. 2017;13(1): e1006507. doi: 10.1371/journal.pgen.1006507 28072817 PMC5224980

[60] Siju KP, De Backer JF, Grunwald Kadow IC. Dopamine modulation of sensory processing and adaptive behavior in flies. Cell Tissue Res. 2021;383(1): 207–225. doi: 10.1007/s00441-020-03371-x 33515291 PMC7873103

[61] Matsuno M, Horiuchi J, Ofusa K, Masuda T, Saitoe M. Inhibiting Glutamate Activity during Consolidation Suppresses Age-Related Long-Term Memory Impairment in Drosophila. iScience. 2019;15: 55–65. doi: 10.1016/j.isci.2019.04.014 31030182 PMC6487374

[62] Hou X, Hayashi R, Itoh M, Tonoki A. Small-molecule screening in aged Drosophila identifies mGluR as a regulator of age-related sleep impairment. Sleep. 2023;46(5): zsad018. doi: 10.1093/sleep/zsad018 36721967

[63] Peek MY, Card GM. Comparative approaches to escape. Curr Opin Neurobiol. 2016;41: 167–173. doi: 10.1016/j.conb.2016.09.012 27710794

[64] Yamamoto D, Koganezawa M. Genes and circuits of courtship behaviour in *Drosophila* males. Nat Rev Neurosci. 2013;14(10): 681–92. doi: 10.1038/nrn3567 24052176

