# Supplementary figures and images for: Biological aging of two innate behaviors of Drosophila melanogaster: Escape climbing versus courtship learning and memory
Source: PLoS One. 2024 Apr 9;19(4):e0293252. doi: 10.1371/journal.pone.0293252 (PMC11003613; doi:10.1371/journal.pone.0293252)

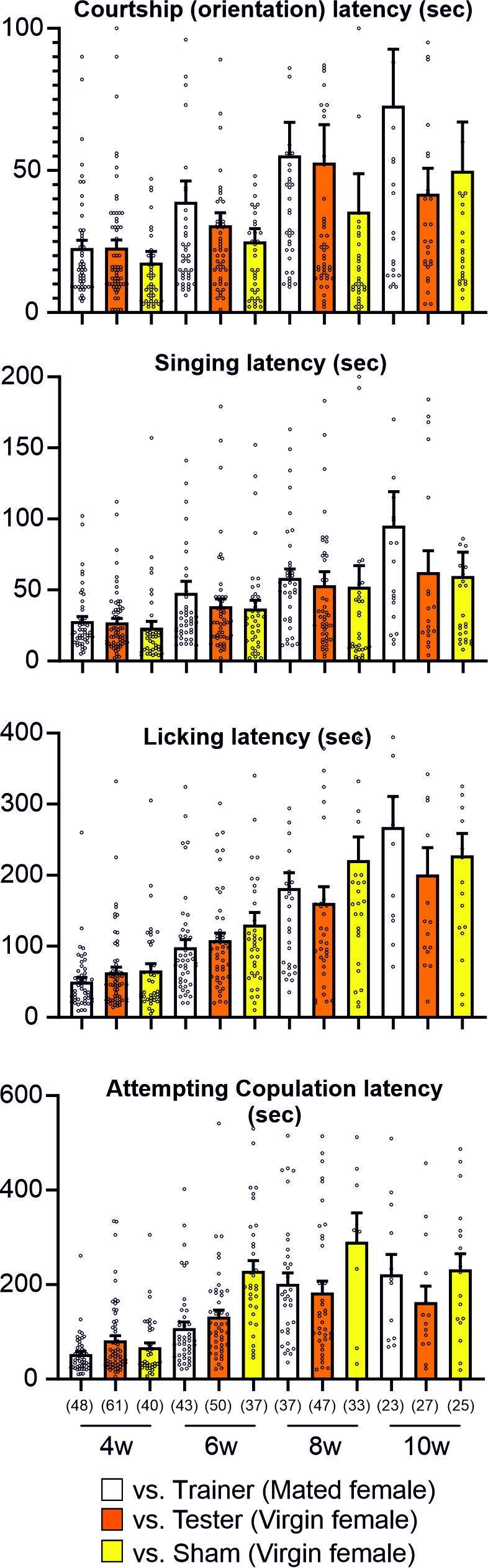

Supplement: S1 Fig — (JPG) [file pone.0293252.s001.jpg]

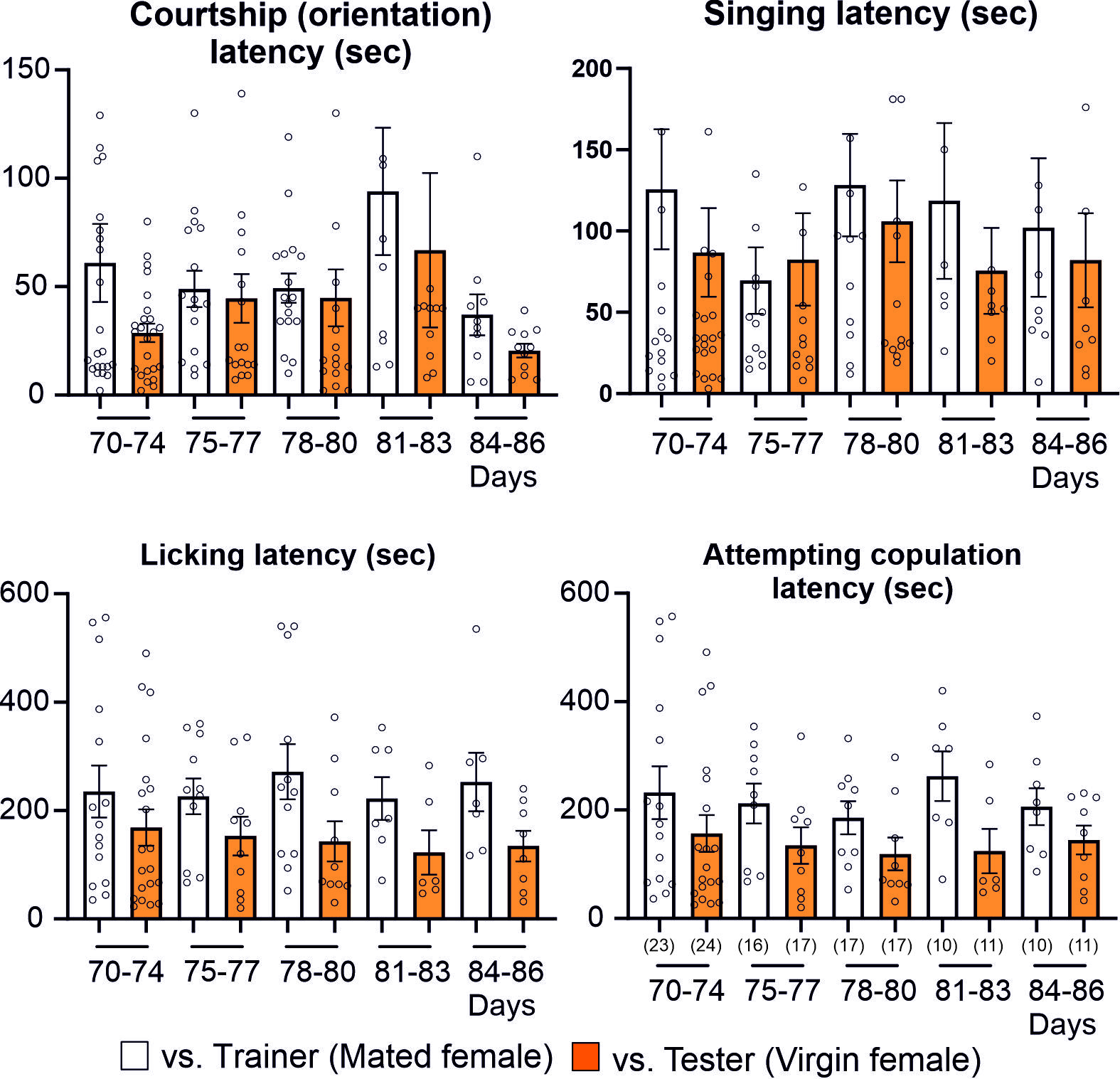

Supplement: S2 Fig — (JPG) [file pone.0293252.s002.jpg]
